# Benefits of Incorporating Lignin into Starch-Based Films: A Brief Review

**DOI:** 10.3390/polym16162285

**Published:** 2024-08-13

**Authors:** Lamia Zuniga Linan, Farayde Matta Fakhouri, Gislaine Ferreira Nogueira, Justin Zoppe, José Ignacio Velasco

**Affiliations:** 1 Department of Chemical Engineering, Federal University of Maranhão (COEQ/UFMA), Av. dos Portugueses 1966, São Luis 65080-805, Brazil; 2 Poly2 Group, Department of Materials Science and Engineering, Universitat Politècnica de Catalunya (UPC Barcelona Tech), Carrer de Colom 11, 08222 Terrassa-Barcelona, Spain; justin.zoppe@upc.edu (J.Z.); jose.ignacio.velasco@upc.edu (J.I.V.); 3 Department of Biomedical and Health Sciences, Minas Gerais State University, Passos 37900-106, Brazil; gislainefnogueira@gmail.com

**Keywords:** biopolymer, mechanical properties, barrier properties, thermal properties, biological degradation, hydrophobic properties

## Abstract

Polysaccharides are an excellent renewable source for developing food-packing materials. It is expected that these packages can be an efficient barrier against oxygen; can reduce lipid peroxidation, and can retain the natural aroma of a food commodity. Starch has tremendous potential to be explored in the preparation of food packaging; however, due to their high hydrophilic nature, packaging films produced from starch possess poor protective moisture barriers and low mechanical properties. This scenario limits their applications, especially in humid conditions. In contrast, lignin’s highly complex aromatic hetero-polymer network of phenylpropane units is known to play a filler role in polysaccharide films. Moreover, lignin can limit the biodegradability of polysaccharides films by a physical barrier, mainly, and by non-productive bindings. The main interactions affecting lignin non-productive bindings are hydrophobic interactions, electrostatic interactions, and hydrogen-bonding interactions, which are dependent on the total phenolic –OH and –COOH content in its chemical structure. In this review, the use of lignin as a reinforcement to improve the biodegradability of starch-based films in wet environments is presented. Moreover, the characteristics of the used lignins, the mechanisms of molecular interaction among these materials, and the sensitive physicochemical parameters for biodegradability detection are related.

## 1. Introduction

Environmental concerns regarding the dependency on petroleum resources over the past decades have fostered significant investments in research and development to minimize the impact of non-renewable resources. This trend is probably going to continue as more and more businesses and consumers seek out sustainable alternatives and become environmentally conscious. As an eco-friendly substitute for plastic packaging, starch-based packaging has seen a rise in popularity in recent years. The starch-based packaging market size is projected to be valued at US$ 7.2 billion in 2023 and is expected to rise to US$ 13.4 billion by 2033. Furthermore, Future Market Insights [1] projects that between 2023 and 2033, sales of starch-based packaging will increase at a noteworthy compound annual growth rate (CAGR) of 6.4%. The biodegradability, compostability, and adaptability of starch-based packaging are reasons for this market demand. However, some weaknesses prevent packaging made of starch to meet a broad range of engineering applications. Due to its polyol and semi-crystalline structure, starch-based packaging is sensitive to water conditions and has poor mechanical properties and high kinetics of enzymatic biodegradability, which is proportional to the starch content [2,3]. As a result, plastic packaging has a longer shelf life than packaging made of starch.

However, technological advancements in the manufacturing of starch-based products are estimated to boost improvements in their applications. It is known that edible coatings derived from natural components such as chitosan and gelatin can improve the barrier quality of starch film, thus extending the shelf life of food products [4]. The superhydrophobic starch-based packaging material are an example of this, specifically the nano-starch/poly-(dimethylsiloxane) coating related by Yateng Wang et al. [5].

On the other hand, nanotechnology is being explored in order to enhance the resistance to water, oxygen, and other gases, as well as to improve the mechanical strength of starch-based packaging. Moreover, biodegradable additives such as polylactic acid, polyhydroxyalkanoates, and polybutylene adipate terephthalate are being researched to enhance the biodegradability of the starch-based films [6]. 

Accordingly, starch and lignin are abundant natural polymers with particular properties that have garnered attention throughout the last decade in the field of bioplastics for having demonstrated the potential to be used in structural engineering. These naturally abundant materials can compete with petroleum-based plastics in both cost and performance to increasingly diminish the dependency of this fossil source [7].

Sufficient works have shown that these two materials can be combined to form a strengthened bioplastic, with barrier and mechanical properties superior or lower depending on the content of synthetic additives. To produce a more hydrophobic material, lignins could be added to the starch-based packaging formulation as a viable substitute.

As it functions inside the wood cell wall, i.e., as an adhesive to combine the cellulose and hemicellulose, lignin is used mostly as a binder in the chemical industry, alongside other traditional applications (byproduct of chemical pulping that is burned in pulp mill boilers for the recovery of pulping chemicals). Additionally, due to its thermoplastic property (which typically ranges from 90 to 170 °C) [8], its aromaticity, its highly cross-linked structure, and its functional groups, lignin is quite reactive and is able to interact with many polymers and change their wettability and enhance mechanical properties and antioxidant activity, among other advantages [9].

This review presents the recent developments regarding the use of lignin as an additive or reinforcement to improve the biodegradability of starch-based films and the physicochemical mechanisms (bonds, molecular interactions) that participate in the final film properties. Additionally, this review describes the properties of the lignins used, the main characterization techniques, and the most critical parameters involved in the material biodegradation.

## 2. Food Packaging Made of Starch

Having a chemical formula of (C_6_H_10_O_5_)_n_, starch is a naturally occurring polymer made up of lengthy chains of glucose molecules joined by glycosidic bonds. Amylose and amylopectin are the two primary polymeric constituents that make up starch at the molecular level. The properties of these constituents vary based on their ratio, which is determined by the source [10]. Starch is found in the form of granules of 0.1 to 200 μm, which are insoluble in cold water but highly sensitive to the heat. Starch granules experience different stages of structural organization during thermal and non-thermal processing in excess of water [11]. Although starch is insoluble in cold water, it can absorb up to 30% wt. The low temperature heating causes the starch granules to expand, but the crystalline portion of the granules stays intact. This stage is termed amylose leaching. The second stage is the gelatinization, which is characterized by the formation of a hydrophilic colloidal solution [12], as a result of the continuous heating of the starch solution. In this stage, the starch granules are disrupted due to the swelling of the crystalline regions. Finally, the retrogradation, which occurs when the starch solution is cooled to a sufficiently low temperature, promotes the reformation of hydrogen bonds, and ordered structures are reestablished; at the same time, the viscosity of the starch solution increases and the gel is formed [13].

Because starch is inexpensive, naturally occurring, non-toxic, renewable, and biocompatible and can form films, it is a material that is extensively researched and utilized in the production of biodegradable films. Furthermore, starch’s polyhydroxy nature makes it simple to modify its structure and functional characteristics through enzymatic or chemical means [14].

Due to these qualities, starch is a material that shows promise for use in food packaging. Enclosing food to prevent tampering or contamination from physical, chemical, and biological sources is known as food packaging [15]. Traditionally, food packaging is produced from petroleum-based plastics (mainly, polyethylene), which, despite having excellent barrier and mechanical properties, due to their non-biodegradability, lead to a substantial ecological burden on the planet. 

More precisely, plastic particles can severely pollute the environment and jeopardize human health by entering the ecological cycle through the water cycle [2,16,17].

In order to overcome this inconvenience, and with the collaborative effort of material science, food science, information science, and socioeconomics, in the past ten years, the rapid development of food packaging from biodegradable polymers of agricultural and lignocellulosic sources, such as polysaccharides, lipids, vegetable and animal proteins, straw, husks, and wood has become evident.

Starch is a common polysaccharide for the preparation of food packaging due to its abundant yield, promising sustainability and excellent biodegradability. Starch-based packaging has been produced, mainly in the form of edible starch-based film, edible fruit and vegetable surface coating, starch-based aerogel material, and starch-based foam packaging materials [2].

Several works have supported the excellent capability of starch to integrate natural supplements to improve the properties of the films. Using white, red, and black rice starch, da Silva, Velasco, and Fakhouri [18] created bioactive starch-based films and investigated the effects of plasticizer, concentration, and starch source on the properties of the films. The authors discovered that variations in opacity, thickness, water solubility, and water vapor permeability were caused by changes in these parameters. Moreover, films with compositions of 5% red or black rice starch and 30% sorbitol could be considered bioactive packaging due their antioxidant capacity, which is transmitted by phenolic compounds. 

With the objective to introduce antioxidant capacity, Malherbi at al. [19] included acai pulp (*Euterpe oleracea* Mart.) extract in films of cassava starch to be used as olive oil sachet packaging. The study showed that films with 1% to 3% extracted acai maintained the stability of olive oil; moreover, the mechanical properties of the film were improved. However, some specific parameters such as peroxide index (IP) and acidity index (IA) did not satisfy the limits established by the current legislation. The authors believe that the presence of oxygen in the headspace of the packaging and the phenolic compounds present in acai extract (which act as free radical scavengers) could trigger the first stages of lipid oxidation.

Nogueira, Fakhouri, and Oliveira [20] and Nogueira et al. [21] incorporated blackberry powder in arrowroot starch films and evaluated its effect on the physicochemical properties of the films. The films displayed increased elongation at break, thickness, water solubility, and water vapor permeability. Moreover, the blackberry particles communicated bioactive compounds and antioxidant capacity and color; even so, in every formulation, the tensile stress dropped (from 0 to 40%, mass/mass of dry starch). The authors claim that certain components of blackberry pulp, such as proteins, fibers, lipids, sugars, and bioactive compounds, cause discontinuities in the polymeric matrix and are responsible for the decline in mechanical properties.

Rodrigues et al. [22] tested the antimicrobial activity of copaiba oil in *Xanthosoma mafaffa Schott* starch-base films and found that the copaiba oil, especially in microencapsulated form, was able to inhibit the tested bacteria (*Bacillus subtilis* and *Staphylococcus aureus*), and the films showed inhibition zones higher than the control. Copaiba oil additionally improved the films’ elongation and tensile strength and reduced the transmittance. Additionally, the oil increased contact angles and decreased solubility, indicating its potential as an additive in the preparation of starch-based food packaging. 

Freitas et al. [23] found that sorbitol plasticizer promoted the low permeability to water vapor in formulations of 20 and 30% (mass of sorbitol/mass of dry starch) in films produced with pea starch and active compounds present in purple araçá (*Psidium myrtoides*). Additionally, the formulation inhibited the growth of *Staphylococcus aureus*; thus, purple araçá and sorbitol were demonstrated to be potential alternatives for the production of antimicrobial food packaging.

Nogueira et al. [24] concluded that arrowroot starch films with grape pomace extract have great potential to be consumed as food packaging or fruit strips. The authors verified the presence of intermolecular interactions between the polymer chain and the additives (such as starch-grape pomace extract, starch-water, sugar-starch, and soluble fiber-starch), evidenced by the improved solubility of the films in water. 

Considering the above, even though properties of starch-based food packaging have been in continuous improvement for extending engineering applications, water barrier and mechanical properties continue to be their main limitation. This is because native starches have poor mechanical properties and are extremely sensitive to water. Moreover, their melting points are higher than the thermal degradation temperature, which challenges their industrial processing (spinning, extraction, and printing technologies, for instance) [2]. 

In particular, the water sensitivity of starch promotes the biodegradability of the food packaging in a proportion related to the content of starch, i.e., the higher the starch content, the higher the biodegradation rate [25]. Water promotes the decomposition of starch-food packaging by destroying hydrogen bonds, which generates a poorly bonded structure [2].

Kaur et al. [26] state that a material’s biodegradability is determined by its capacity to break down in the presence of environmental factors, specifically the enzymatic activity of microorganisms like bacteria and fungus. According to Cheng et al. [14], there are two common methods for estimating the biodegradability of starch-based films: (a) weight loss, which involves burying film samples in the ground, removing them after a while, cleaning them with water, and drying them at 4–5 °C. The final weight loss of the specimens is then measured [26]. (b) Periodically, the weights of a carbon dioxide (soda lime) and a water (CaCl_2_) absorption column are measured to estimate the amount of CO_2_ released by the soil microbial community [27].

Li et al. [28] analyzed the effect of different structures on enzymatic degradation of starch films. For the study, the authors considered the degradation of normal maize starch by fungal α-amylase of samples with low molecular weight and molecular size and samples of higher crystallinity and monitored the degradation kinetics of the compression molded starch films. Thus, it was concluded the degradation of the films occurs in two stages. In the first stage, degradation of easily accessible components, such as small molecules that enter in the solution, are rapidly degraded. In the second stage, the highly disordered and accessible chains at the films’ surface degrade. Both stages follow a simple first-order kinetic degradation. At this point, the authors noticed that a resistant structure formed during retrogradation, significantly reducing the degradation rate.

The introduction of lignin in starch films has been envisaged as a promising alternative to overcome both structural weakness and water resistance, because, in nature, lignin protects polysaccharides from hydrolytic enzymes.

## 3. Lignin

Lignin is a natural, renewable, and amphiphilic polymer derived from hydroxycinnamyl alcohols, p-coumaryl, coniferyl, and sinapyl and its functional groups hydroxyl, carboxyl acid, methoxyl, ether, and ester, produced by a dehydrogenating polymerization involving radical coupling [29]. It is a three-dimensional polymer without any sugar in it. Because of its extremely intricate aromatic hetero-polymer network of phenylpropane units, it can be used as a filler in conjunction with polysaccharide films. All plant fibers are composite structures in large part cellulose but with an important proportion of hemicellulose and lignin. Cellulose is the main structural component (load-bearing), while hemicellulose/lignin matrix gives flexibility, controls the moisture content, and protects against pathogens. Furthermore, lignin forms lignin carbohydrate complexes in plant cell walls by physically and chemically attaching to cellulose and hemicellulose through covalent bonds like benzyl-ether, benzyl-ester, and phenyl-glycoside bonds [7]. 

**Figure 1:**
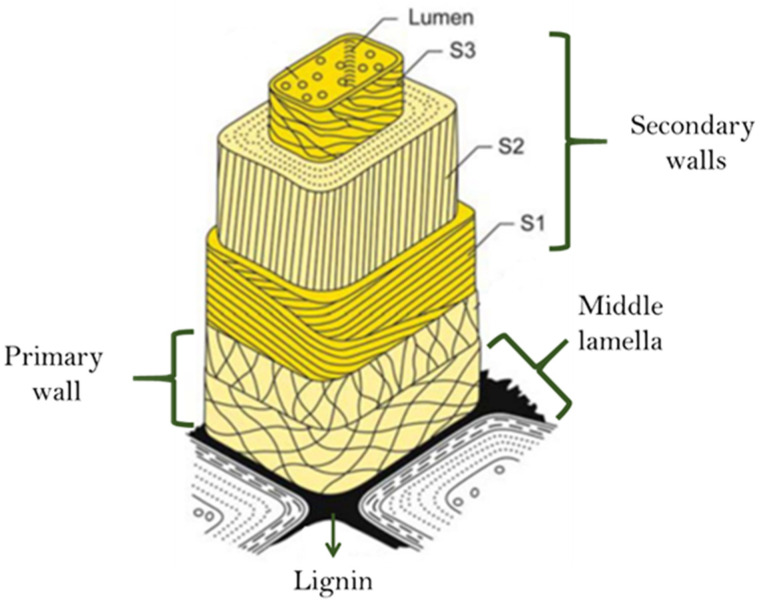
Distribution of lignin in wood cell walls. Adapted with permission from Sjöström, 1993 [30], copyright 30 July 2024 Elsevier license No 5838970067490.

In Figure 1, Sjöström [30] presents the structural composition of the cell wall of the wood cell wall, where the middle lamella is enriched in lignin [31]. The secondary walls contain the largest proportion of the total lignin in the cell walls.

Lignin has been reported to possess environmentally and industrially useful properties such as high degree of functionality, better adhesiveness, and adsorption and solution properties, along with good biocompatibility, low toxicity, and antibacterial properties. 

**Figure 2:**
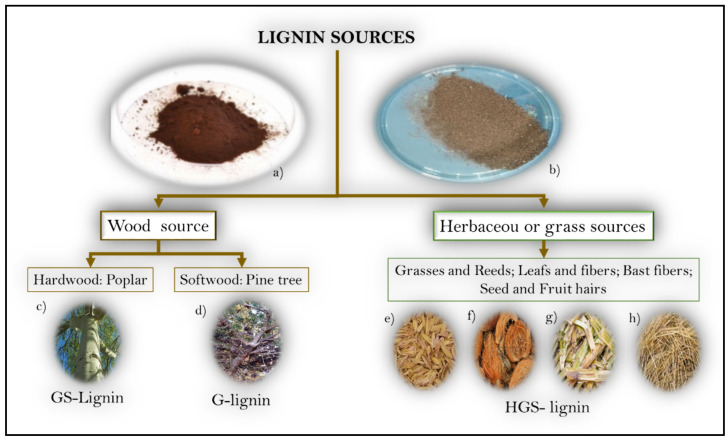
Lignin classification according to the precursor. Font: (**a**) https://www.infoholicresearch.com/report/kraft-lignin-market/, accessed on 30 July 2024; (**b**) Zuniga Linan et al. [32]; (**c**) https://tree-pictures.com/images/treephotos-poplar/poplartree/poplar-tree-bark.jpg, accessed on 30 July 2024; (**d**) The authors; (**e**) https://stock.adobe.com/es/search?k=%22rice%20husk%22, accessed on 30 July 2024; (**f**) https://pt.dreamstime.com/foto-de-stock-casca-seca-do-coco-image67395503, accessed on 30 July 2024; (**g**) Font: https://www.revistarural.com.br/2020/06/02/pesquisa-permite-gerar-etanol-a-partir-de-bagaco-de-cana/, accessed on 30 July 2024; (**h**) Font: https://pt.dreamstime.com/fundo-da-palha-do-arroz-image108812076, accessed on 30 July 2024.

The classification of lignins considers the source precursor and the extraction methods. According to the precursor, in Figure 2, it is possible to identify the wood lignins, extracted as a byproduct of processing of hardwood (Angiosperm dicotyledons) or softwood (Gymnosperms) in the pulp and paper industry, and the herbaceous or grass lignins, which are extracted from annual plants (Angiosperm monocotyledonous).

According to the extraction method, several lignins are identified as follows:

### 3.1. Kraft

It is a byproduct of the alkaline pulping process, where the biomass is treated between 140 and 180 °C with an aqueous mixture solution of sodium hydroxide and sodium sulfide for 2 to 4 h. Due to high temperatures and alkaline conditions, linkages between the lignin units (β-O-4’) were disrupted, and some degradation occurred. Kraft lignin has a higher concentration of condensed C-C structure and phenolic –OH groups [33] than native lignin. Softwood kraft lignin appears to contain between 60 and 70 phenolic hydroxyl groups for every 100 aromatic rings. This can be recalculated to 3.6 to 4.2 mmol/g of lignin if one assumes that a phenylpropane unit has an average molecular weight of 168 [34,35]. Low molecular weight lignin, on the other hand, is thought to have a much higher frequency; most lignins have a value of roughly 45–60 phenolic units per 100 aromatic rings (2.7–3.6 mmol/g lignin) [34]. Based on the unit in which they are found, phenolic hydroxyl groups can be classified into syringyl, non-condensed and condensed guaiacyl, and p-hydroxyphenyl. The aliphatic OH content of the acid kraft lignin samples was 1.11 mmol and the phenolics OH content was 2.28 mmol/g of syringyl, 1.11 mmol/g of condensed guaiacyl, 0.99 mmol/g of uncondensed guaiacyl, and 0.13 mmol/g of p-hydroxy phenyl, totaling 4.50 mmol/g of total phenolics content. The phenolics OH content of alkali kraft lignin samples was 2.05 mmol/g for syringyl, 1.07 mmol/g for condensed guaiacyl, 0.9 mmol/g for uncondensed guaiacyl, and 0.13 mmol/g for p-hydroxy phenyl, with a total of 4.14 mmol/g for phenolics. In contrast, the aliphatic OH content of the samples was 1.00 mmol/g [36].

### 3.2. Organosolv Lignins

Organosolv lignins are extracted through pure solvent, an aqueous solution (mixture 1:1), or a solution of organic solvents, such as methanol, ethanol, formic acid, and acetic acid. Organosolv lignins are highlighted because of their low molecular weight and polydispersion. In addition, these contain more phenolic -OH groups and are sulfur-free compared to kraft lignin. In particular, Fang et al. [33] affirmed that these characteristics are convenient for efficient transformation of lignins into carbon fibers. An example of this is the Alcell lignin, which was found to be appropriated for the production of carbon nanofibers by electrospinning due to the low contents of inorganic compounds.

### 3.3. Formic Acid Lignin

Formic acid lignin is a kind of organosolv lignin made by formic acid fractionation-based selective lignocel-lulosic biomass separation. According to Ni et al. [37] and Zhang et al. [38], this lignin, which is prepared at high pressure (i.e., above the atmospheric pressure) and high temperature (e.g., 145 °C) presented less phenolic and aliphatic hydroxyl groups (specifically, the phenolic hydroxyl group on the p-hydroxyl units), which are responsible for the lower water repellence. On the other hand, in this lignin, the structural units of syringyl (S) and guaiacyl (G) increase, and for this reason, the amount of hydrophobic methoxy groups increases as well.

### 3.4. Soda/AQ

This lignin is produced by pulping soda-anthraquinone, a sulfur-free method. The biomass—mostly annual plants with a small amount of hardwood plants—is treated with an aqueous sodium hydroxide solution at 160 °C [33].

### 3.5. Alkali

Alkali is a product of hydrolytic cleavage of native lignin, mainly from annual plants such as flax, bagasse, straw, and husk and with some limitations from hardwood [39].

### 3.6. Lignosulfonates (LSs)

Sulfonated lignins are water-soluble anionic polyelectrolyte polymers that possess higher molecular weight than alkali lignin. Both alkali and lignosulfonates are derived from the pulping process (sulfite pulping) in papermaking and are commercially available [39,40].

### 3.7. Hydrotrope Lignin

This lignin has been extracted by using hydrotropes, which are a class of amphiphilic molecules that promote the aqueous solubility of organic molecules by mediating the interactions between hydrophobic and hydrophilic molecules but which are unable to form well-organized structures in water (such as micelles). Examples of hydrotropes are p-ter-butylbenzenesulfonate, sodium cumenesulfonate, sodium p-toluenesulfonate, 3,4-dimethylbenzenesulfonate, and p-Toluene sulfonic acid (p-TsOH). The last one was used by [41] to extract hydrotropic lignin from rice straw.

### 3.8. DES Lignins

These types of lignins are extracted using deep eutectic solvents (DES) in the pretreatment stage of the delignification process. The DES lignins are a new generation of green solvents with characteristics of both ionic liquids and organic solvents. Thus, the extraction is cheaper and the lignin does not need purification [7]. Examples of DES are the combination of (lactic/acid/glycerol/choline chloride) used for delignification of wheat straw [42] and (lactic acid/betaine) used by Guo et al. [43] for pretreating poplar biomass. 

### 3.9. LignoBoost Lignin

The lignin in question is the solid biofuel that the pulp and paper industries recover from the kraft lignin stream. Thus, the lignin is precipitated from the black liquor by acidification and filtered, producing a cake, which is dispersed and acidified. From the resulting slurry, a lignin with higher yield and less ash and carbohydrate contents is obtained [44,45].

### 3.10. Lignin Nanoparticle (LNP)

Lignin has the potential to be used in advanced chemistry, as it is one of the most promising sources to fabricate carbon materials. However, even though its potential is being recognized, this material is still underutilized, as its major application is as a fuel source. 

Especially as a reinforcement in polymeric composites, crude lignin offers some drawbacks, such as repulsive interaction in the interphase that decreases the mechanical properties of the final product. When lignin reaches the nanoscale, its surface and other characteristics are improved, and its applications can be further expanded [46]. Specifically, [47] demonstrated that the insolubility and indigestibility of lignin could be overcome if it is transformed into spherical microparticles of 0.1–3 µm in diameter. Because of this, lignin in nanoscale size or lignin nanoparticles (LNPs) emerged, and its preparation methods from the black liquor of wood pulping or from plain lignin have been improved in terms of simplicity and sensitivity toward the environment. 

**Figure 3:**
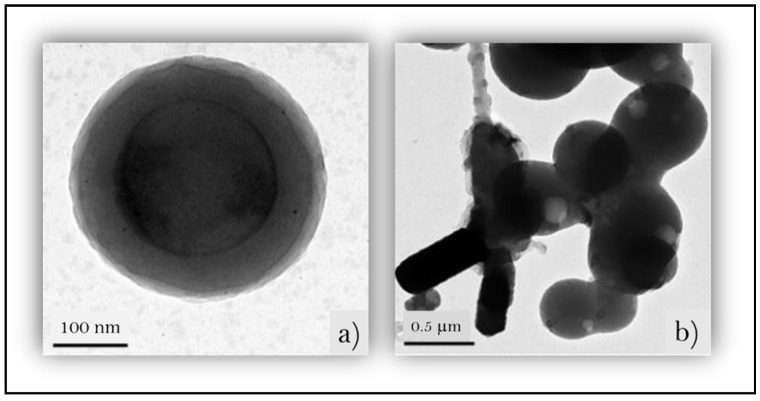
“Core-shell” structure of a lignin nanoparticle (**a**) and the cluster aggregation of LNP spheres (**b**). Adapted with permission from SHUZHEN NI, HUIYANG BIAN, YONGCHAO ZHANG, et al., 2022 [37]. Copyright, 7 July 2024 American Chemical Society).

According to Ni et al. [37], the hydrophilic portion of LNP is oriented toward its surface, while the hydrophobic phenylpropanoid units have self-assembled into the core of this spherical particle, which is known as the “core-shell.” Figure 3a shows the “core-shell” configuration of an LNP prepared through the dissolution of one gram of formic acid lignin in 50 mL of tetrahydrofuran under constant stirring (500 rpm) for one hour. Figure 3b is the image of several individual lignin nanoparticles (LNPs) forming the clustered agglomerates with each other in three or more LNP spheres. The authors incorporated such LNPs to corn starch films to enhance hydrophobicity. 

**Figure 4:**
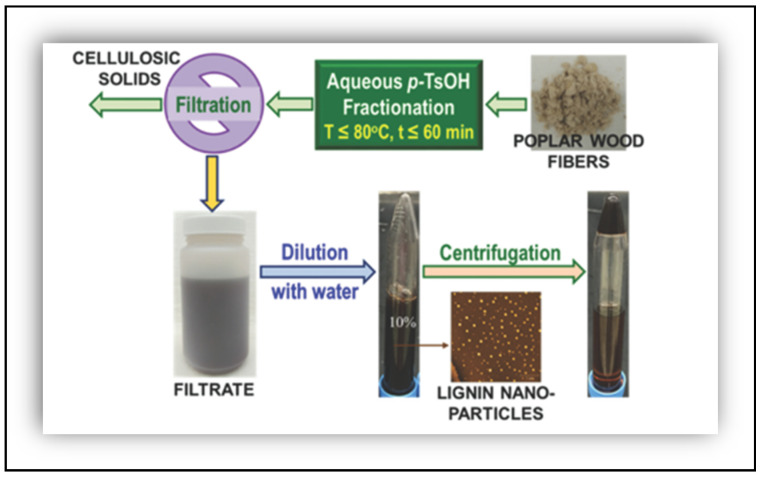
Flow diagram of preparation of LNPs from the black liquor extracted with the p-TsOH method. Reused with permission from Ma et al., 2018 [48]. Copyright 08 August 2024 DE GRUYTER.

Figure 4 describes the flow diagram of an economic and simple fractioning of poplar wood, using p-toluenesulfonic acid (p-TsOH) to produce LNP in an aqueous system at temperatures under 80 °C and less than 60 min. After extracting the liquor, it was diluted with distillated water and the resultant liquor was filtrated under vacuum to separate the water-insoluble solids. The filtrate was newly diluted (10%) and centrifuged for 10 min, where the precipitated phase formed the LNPs [48].

### 3.11. General Characteristics of Lignin

The rigid, heterogeneous, and three-dimensional structure of lignin is formed during the enzymatic dehydrogenation of its precursor alcohols p-coumaryl, coniferyl, and sinapyl. The predominant bonds are C-O and C-C; however, additional components of lignin such as hydroxycinnamic acids and flavonoids render the structure more complex, with additional linkages and functional groups [49].

P-coumaryl, coniferyl, and sinapyl alcohols separately form “larger repeat units” during the lignin synthesis process, according to a phenylpropane mechanism. These correspond to p-hydroxyphenyl (H), guaiacyl (G), syringyl (S), and caffeyl alcohol (C), with the last one being the most recently identified [50]. These units are mainly linked by β-O-4 bonds.

A complete structural model of lignin is still under debate. Traditional techniques such as infrared spectroscopy (IR) and advanced techniques such as two-dimensional nuclear magnetic resonance (NMR) and heteronuclear single quantum conference (HSQC) have allowed the identification of functional groups and quantification of lengths, branching and predominant interunit linkages.

**Figure 5:**
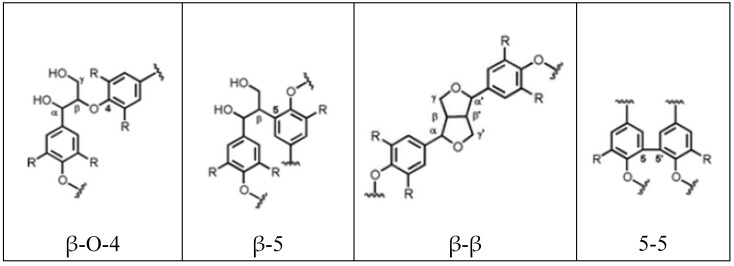
Structures of most abundant linkages in lignin.

**Figure 6:**
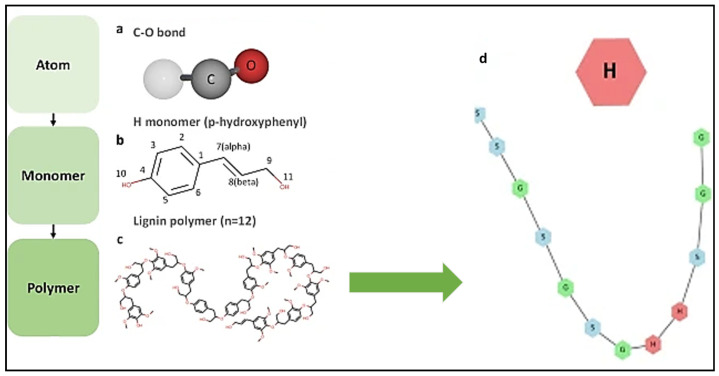
Multiscale structural representation of the HGS lignin built through LigninGraphs software. (**a**) C-O bond representation; (**b**) H monomer; (**c**) lignin polymer of 12 monomers; (**d**) big graph for the lignin polymer, where each monomer is a node, and each linkage is an edge. The large repeat units are represented in colorful hexagons, blue (S-unit), green (G-unit), and red (H-unit). Adapted with permission from Wang et al., 2022 [52]. Copyright, 24 July 2024 Springer Nature.

Computational models have shown advances in the identification of the lignin structure [51,52]. In particular, Yifan Wang et al. [52] created a model that, using data from experiments, built lignin structure libraries using a multiscale graph-based modeling framework (LigninGraphs). The authors did an accuracy determination of percentages of repeat units, bonds, and molecular weight for hardwood (poplar), softwood (pine), and herbaceous (miscanthus). Some representative data are summarized in Table 1 below, and Figure 5 shows the structures of more abundant linkages β-O-4, β-5, β-β, and 5-5. Figure 6 is the representation of the lignin polymer built through LigninGraphs.

The influence of the type of solvents and operational conditions (temperature, time, and pressure) is principally on the ratio of phenolic units (H, G, and S) and on the content of residual compounds (purity), thereby interfering with the thermal and mechanical properties of lignins.

For example, the G unit makes up the majority of the lignin in softwood, whereas the G and S units are present in lignin from hardwood. In contrast, grass lignins are more complex in structure, as they are rich in three phenylpropane units, H, G, and S. The results of [55] showed that lignins from different source precursors, extracted by the same method, had different proportions among their phenolic units H, G, and S. For example, the H/G/S molar ratio of organosolv lignin from corn residue is 36/34/30, which is far distant from 4/33/63 of organosolv lignin from hardwood (Aldrich). In addition, organosolv extraction using microwave irradiation promotes lignin depolymerization and reduces the formation of condensation products.

Softwood lignins have higher molecular weight and polydispersity than hardwood lignins, while herbaceous organosolv lignins have lower molecular weight and polydispersity than wood lignins. The Mw of grass-based organosolv lignins varies between 2000 and 4000 g/mol, whereas the Mw of wood-based kraft lignins ranges from 20,000 to 40,000 g/mol [33]. In addition, the quantification of the molecular weight of organosolv lignins is more reliable, given that these lignins are completely soluble, which means that acetylation is not necessary for this purpose. Thus, conventional techniques, such as GPC chromatography with solvents (THF) and fractioning columns and detectors can be used, representing minor time and cost savings. With regard to the solubility, independently of the source, lignins that are soluble in organic solvents have lower molecular weight and polydispersity index (PDI) than insoluble lignin. Moreover, –OH phenolic groups are more abundant in low-molecular-weight lignins, while –OH aliphatic groups are more present in high-molecular-weight lignins [56].

The effect of the extraction method on the chemical composition of lignin was also evidenced by the FTIR spectra of soda lignin [57,58] and an organosolv lignin [59] from wheat straw, in which soda lignin had higher content of carboxylic groups compared to the organosolv lignin. 

With regard to the response of lignin during its application, studies have so far demonstrated that herbaceous-based lignins with higher S phenolic content tend to be more stable below 250 °C, which is advantageous for use as a photostabilizer in polyolefins against light or thermal oxidation [55,60]. Softwood lignins with higher levels of G units than H units show a higher intensity of hydrogen bonds due to the presence of aliphatic groups (carbon–carbon bonds), which corresponds to more rigid structure. This characteristic leads to higher Tg values that hinder the modification, blending, and thermal treatment stages during the manufacture of LCFs (lignin-based carbon fibers) [33]. However, the same characteristic renders these lignins with higher carbon yields and the facilitation of stabilization. Therefore, compared to hardwood lignin-based carbon fibers, softwood lignin-based carbon fibers can be stabilized 26 times faster. Fang et al. [33] showed that kraft lignins of softwood have higher aliphatic –OH groups than hardwood kraft lignins, whereas the opposite tendency is found for phenolic –OH groups. The presence of these groups influences the formation of the intra- and intermolecular hydrogen bonds of lignins (i.e., aliphatic groups form strong intermolecular bonds and phenolic –OH groups form intramolecular bonds with methoxyl groups). This explains the fact that the softwood lignins have more rigid structures and higher Tg.

On the other hand, Monteil-Rivera et al. [55] highlighted that soda lignin has better performance for applications where high proportions of carboxylic groups are required (chelating agents). Furthermore, the extraction conditions can be adjusted to change the number of aliphatic -OH groups in lignin. If more aliphatic -OH groups are required, milder conditions (temperature and alcohol concentration) in the organosolv process could be used. Therefore, regarding the performance of lignins in engineering applications, some trends can be predicted, but there is not a definitive behavior. The following sections are intended to guide the reader on the characteristics of lignins that have demonstrated good performance in improving the starch-based films’ properties, including but not limited to hydrophobicity. 

## 4. Lignins as a Reinforcement of Biopolymer Films to Improve Their Properties

Considering the goal of broadening the application under humid conditions, relevant contributions have shown routes to prepare polysaccharide-based films, with biodegradation controlled by lignin. The studies have demonstrated that the majority of lignins have promising performance for protection against oxidation, UV radiation, and moisture, especially those with high content of phenolic –OH groups from syringyl and guaiacyl, such as soda and organosolv lignins. Quantitatively, Kim and Hum [61] revealed in their study that the total phenolic OH content of organosolv lignins (OL) was higher than that of soda lignins (SL) (OL: 4.55–6.21, SL: 3.57–4.84 mmol g^−1^, C5-substituted + G + H). On the other hand, the amount of aliphatic OH found in organosolv lignins (OL30: 0.70, OL40:0.47, OL50: 0.60, OL60: 0.57 mmol g^−1^) was two to three times lower than that found in soda lignins (SL1: 1.70, SL2: 1.56, SL4: 1.73, SL6:1.32 mmol g^−1^) [61].

Ni et al. [37] built starch-based composite films with “core-shell” nanoparticles of lignins, which were prepared from isolated lignin from the effluent of formic acid pulping. The as-prepared LNPs, according to the authors, have a great potential for strengthening the hydrophobicity of starch films because they can assemble and form a relatively hydrophobic cluster aggregation. An important result obtained from this work was that after adding the LNPs to the starch films, the water contact angle enhanced from 65° (that of pure starch film) to 98° and 118° (at 1% and 3% LNP content, respectively). This behavior was linked to the addition of cluster aggregation of LNPs with increased hydrophobic methoxy groups, which resulted in the formation of a tight hydrophobic layer. It was confirmed that a rougher surface equated to a more heterogeneous surface, which led to the uneven dispersion of LNPs that assisted in preventing water from penetrating the interior of the films.

The work of Tian et al. [62] related the fabrication of composites of bacterial cellulose and lignin nanoparticles (BC/LNPS) extracted through soda/anthraquinone, deep eutectic solvent, and organosolv techniques. The authors affirmed that the high content of phenolic –OH groups of organosolv lignin contributed to the formation of hydrogen bonds between LNPs and cellulase enzymes, resulting in the inhibition of enzymatic hydrolysis and reducing the biodegradability. Hence, the lignin created a physical barrier and non-productive binding with enzymes that inhibited the biodegradability. Nevertheless, the composite with soda lignin showed the highest water holding capacity (WHC) due to smaller particle size, which resulted in smaller pores of the composites. Moreover, this behavior was attributed to the fact that soda lignin contributed with more carboxyl groups (-COOH), thus it communicated more hydrophilicity [43,63]. Regarding the composites with DES lignin, the authors affirmed that the ability of DES lignin to retard the biodegradability was limited because this lignin exhibits less content of phenolic –OH [64]. 

The results by Zhao et al. [56] regarding the composite of cellulose nanofibers with lignin (CNF/Starch/lignin) showed diverse behaviors of mechanical and thermal properties of the films, depending on the lignin properties. For example, lignins with low molecular weight (hardwood lignin) led to higher Young´s moduli and mechanical strength due to the small molecules of lignin being able to act as a compatibilizing agent between starch and CNF. Furthermore, lignins with higher levels of phenolic and carboxyl -OH groups raised the Young’s modulus, because of the enhance hydrogen bond formation between lignin and the starch and CNF, which contain abundant-OH groups as well. These results are consistent with those reported by [65], where 1% *w*/*v* of kraft lignin from sugar-cane bagasse increased the Young´s modulus of PLA/lignin/castor oil film by 46% and the Young’s modulus of PMMA/lignin/castor oil film by 38%. Moreover, the thermo-oxidative stability of the films was improved with the lignins with a higher amount of phenols containing saturated side chains (from softwood lignin), which showed the influence of the chemical structure of lignin on this property. 

Guo et al. [43] assessed the antioxidant activity and UV-shielding property of each cellulose-based functional film, reinforced with the poplar wood lignins DES, organosolv, soda/AQ, hydrotrope, and kraft. The authors pointed out that antioxidant activity is positively affected with the –OH phenolic groups of syringyl and guaiacyl, which were responsible for radical scavenging. However, there was little contribution of –OH phenolic groups of p-hydroxyphenyl. Thus, organosolv and soda/AQ lignins showed higher antioxidant activities due to their higher content of syringyl units. Additionally, the phenolic –OH groups of syringyl units were also responsible for blocking ultraviolet light. The authors attributed this behavior to the localization of the methoxy groups on the aromatic rings of the lignin, which disrupted the electron distribution, allowing the –OH phenolic groups to stay in an active state. Finally, the authors concluded that the contribution to both antioxidant activity and UV protection varied from high to low according to the amount of –OH phenolic groups present in the phenyl-propane units in this manner: syringyl > guaiacyl > p-Hydroxyphenyl.

Zadeh, O’Keefe, and Kim [40] studied the relationship between lignin and soy protein isolate (SPI) in films made from these materials during the transglutaminase enzyme treatment. The authors improved covalent cross-linking of proteins by using transglutaminase in the SPI structure to catalyze cross-linking between the -amine group in lysine and the -carboxyamide group on glutamate residues bound to proteins. The research allowed them to highlight several benefits of the lignins in the films. First of all, the alkali and lignosulfonate lignins showed significant radical scavenging activity and strong potential in active packaging structures. Particularly, alkali lignin showed higher radical scavenging activity than lignosulfonate. The author attributed this behavior to the structural and chemical composition differences between these lignins. Thus, alkali lignin has lower molecular weight and higher hydrogen donation ability, perhaps due to –SH groups. Then, due to the lignin’s inherent color, films containing alkali lignin demonstrated a strong UV-blocking ability. By using lignin and enzymatic treatment, these common limitations of biopolymeric films in packing were minimized by improving the mechanical properties and thermal stability of films containing these lignins. Shankar, Readdy, and Rhim [66] studied the effect of lignin content on the barrier properties of agar/lignin films for food packaging materials. The authors found that 1% of lignin in the films was enough to significantly reduce the transmittance values by more than 90%. Chromophoric groups of the lignin would be responsible for the absorbance of UV light. Water vapor permeability (WVP) and moisture content (MC) were others of the profited properties, and thus 10% of lignin in the film reduced the WVP and MC by 27%. The author claims that because of the strong intermolecular interaction between the molecules of lignin and agar, there will be less water molecule permeability through the polymer film and an increased path for water vapor diffusion. In particular, there is strong hydrogen bonding between the free hydroxyl groups, and water could reduce the uptake of more water. Moreover, the reduction of moisture was attributed to the aromatic polyphenolic rings present in the lignin. However, some drawbacks could be evident depending on the type of lignin. For example, because the alkali lignin used to reinforce the films in this research was more hydrophilic than the neat agar, its incorporation increased the hydrophilicity of the film, resulting in a decreased water contact angle in comparison to the pure agar.

Bhat et al. [67] produced sago starch-based biofilms with lignin for the food packaging industry. This research tested a kind of alkali lignin, derived from the black liquor waste (BLW) of the palm oil industry. It was demonstrated that the insertion of 4% of lignin in the films improved their properties, specifically water vapor permeability and water solubility. According to a previous study of Baumberger [68] and their own results, the authors attributed this improvement to the miscibility of phenolic compounds of lignin with the amylose of starch and possible cross-linking between starch and phenolic compounds. 

Majeed et al. [69,70] fabricated starch-based films reinforced with kraft lignin in order to use them as a slow-delivery fertilizer. The authors found that lignin retarded the biodegradability of the films when it is exposed to the soil. Thus, lignin reduced the swelling of the network polymer in contact with the moist soil, also impeding the urea-nitrogen release outside the limited swollen network of the polymer before it started to biodegrade. Moreover, lignin generated a recalcitrant property in the starch, which made it further difficult to release urea-nitrogen. The authors proposed an interesting mechanism that justified the increase of the thermal stability of the films, which involved the formation of thermally stable products of biodegradation, due to the participation of the stilbene of the lignin.

Spiridon et al. [29] evaluated the wettability, heat, and mechanical properties of polylactic acid-based composites reinforced with an acetosolv lignin (from birch wood) and a LignoBoost lignin (from kraft cooking of softwood raw material). The authors found that both lignins increased the Young’s modulus of the composites, even after 600 h of accelerated weathering exposition, which was attributed to the good adhesion between lignins and PLA. Moreover, lignins increased the thermal stability of the polymer, delaying the initial temperature of degradation of the composites. Related to the water resistance, the author demonstrated that PLA-composite with acetosolv lignin kept a higher contact angle than the neat PLA (from 29.2° to 49.8°), specifically, after the combined exposure to temperature, humidity, and UV radiation. 

Chantapet et al. [71] evaluated the effect of kraft lignin on the extrusion processing and on the physical properties of wheat gluten bioplastic. Several relevant results allowed the authors to conclude that kraft lignin acted on the vegetal protein as a radical scavenger, capturing the thiyl radicals formed during the extrusion protein. That is, kraft lignin induces protein depolymerization and its association with the vegetal protein. With this form, it was possible to broaden the extrusion processing window. Regarding the physical properties, the authors found that the tensile strength and Young’s modulus of this bioplastic increased when the kraft lignin content increased in the range of 10 to 30%. However, the elongation at break decreased. Furthermore, because kraft lignin has hydrophobic qualities, adding lignin reduced the bioplastic’s absorption of water.

In view of the above, Table 2 summarizes the types of lignins that have been used in biopolymer films and the improved properties.

## 5. Biodegradability of Starch Films and Mechanisms for Controlling Biodegradation with Lignin

The ability to control the degradation rate of starch films offers great benefits for their application as food packaging materials. In that regard, there are somewhat competing interests, in that starch films or coatings should offer protection of and prolonged shelf-life for packaged foods while at the same time be biodegradable under atmospheric or industrial composting conditions at a later stage in the product life cycle. In general, starch films protect foods from spoilage by acting as a barrier against oxygen, which in turn reduces both oxidative degradation and aerobic degradation of foods by microorganisms [72]. The primary drawback of starch films is their hydrophilicity, due to the presence of numerous hydroxyl groups along the polysaccharide chains, which allows the passage of water, thus rendering films more susceptible to hydrolysis and microbial growth. Therefore, many research efforts are focused on enhancing water resistance by increasing the hydrophobicity of starch films while at the same time not compromising their inherently favorable properties as gas barriers [73]. In this section, a brief overview of starch degradation mechanisms, factors affecting biodegradation, and the application of lignin in controlling the degradation of starch films are presented. 

### 5.1. Degradation Mechanisms

Starch, being a polysaccharide composed of α-glucose subunits joined by glycosidic bonds, is susceptible to degradation by various means, including hydrolysis, often catalyzed by amylolytic enzymes (fungal or bacterial), and oxidation, which can be catalyzed by either UV light exposure or heat [74]. In terms of amylolytic hydrolysis, the primary mechanism of starch degradation is glycosidic bond cleavage (depolymerization), catalyzed by amylase enzymes in the presence of water. Excessive exposure to high temperatures and/or UV radiation, for example, by sunlight, further catalyzes starch degradation via oxidation of C-2, C-3, and C-6 hydroxyl groups to carbonyl groups and further cleavage of glycosidic bonds [75]. The resulting α-glucose after depolymerization of starch can be further metabolized by microorganisms, promoting microbial growth.

### 5.2. Factors Affecting Degradation in Starch Films

As they are composed of two structurally different polysaccharides, linear amylose and branched amylopectin, the degradation behavior of starch films first and foremost depends on the film’s composition, which impacts film morphology. As amylopectin is the starch component consisting of a linear backbone with short chain branches, which are mainly responsible for the double-helical crystalline domains in native starch granules [76], one might suspect that higher amylopectin content would improve starch film barrier properties. However, the retrogradation of starch leads to significant alterations in its crystalline order, in that highly branched amylopectin tends to form amorphous domains, while amylose forms crystalline domains upon cooling. For example, Cano et al. [77] determined that higher amylose content in starch films leads to lower oxygen permeability and water resistance due to the formation of crystalline regions of amylose. Furthermore, compression-molded films of retrograded starch were degraded by α-amylase at a lower rate compared to non-retrograded films, due to the increased structural ordering of amylose chains upon retrogradation [28]. 

The aforementioned studies emphasize the impact of retrogradation on starch film properties, in that re-crystallization of amylose and amylopectin mixtures gives rise to crystalline domains comprised primarily of amylose. Regarding the molecular weights of amylopectin and amylose, which vary depending on the starch source, [78] did not find significant differences in hydration of starch films produced from potato, corn, wheat, or rice (molecular weights ranging from 51 to 83 MDa). In the case of pure amylopectin and amylose films, [79] found that neither varying the degree of crystallinity nor varying the network structure had any impact on water vapor and oxygen permeabilities. 

One of the most common methods to enhance water resistance and alter the degradation rate of starch films is through the incorporation of additives [80]. Such additives include hydrophobic compounds, essential oils, extracts, polymers, plasticizers, inorganic compounds, and antioxidants. For example, the addition of green tea and palm oil extracts to cassava starch films led to a 50-fold decrease in water vapor permeability [81]. The incorporation of lycopene nanocapsules also has a positive impact on water resistance of starch films due to increased hydrophobicity [82]. While there are numerous studies and reviews on the use of such additives in starch films, the application of lignins in controlling starch film degradation remains less explored. In the following section, the mechanisms of action of lignin in controlling the degradation of starch films will be presented.

### 5.3. Lignin Mechanisms of Action in Controlling Degradation

As discussed in previous sections, lignin offers multiple functions in polysaccharide films that positively contribute to the film degradation rate. Those functions include hydrophobicity, UV blocking, plasticization, antimicrobial activity, and oxidation inhibition [83]. A majority of these characteristics are a result of the phenolic chemical structures of the monolignol units, having both hydrophobic (aromatic) and hydrophilic (hydroxyl -OH) functional groups. Moreover, lignin content is well known to have a significant impact on biomass recalcitrance, in that higher lignin content often leads to decreased sugar yields in enzymatic hydrolysis towards ethanol production [84]. In terms of biodegradation, lignin acts as both a physical barrier and a non-productive binding site for enzymes, the latter being mediated by hydrophobic (van der Waals) hydrogen bonding and electrostatic interactions [85]. With respect to hydrogen bonding and electrostatic interactions, the total phenolic -OH content and carboxylic acid -COOH content can often be correlated with the enzymatic hydrolysis rate, in which hydrogen bond formation between lignin and enzymes inhibits hydrolysis [64]. 

As mentioned in Section 5, Tian et al. [62] observed significant differences in enzymatic hydrolysis efficiency of bacterial cellulose films containing ca. 40% of either DES, organosolv, or soda lignin (MW = 1–1.8 kDa), in which non-productive adsorption was dominated by electrostatic repulsion between lignin and cellulase enzymes [62]. In this case, the authors showed that the higher the absolute zeta potential value of lignin, the higher the enzymatic hydrolysis efficiency. Similar mechanisms can be argued for the case of lignin-reinforced starch film degradation by amylases. Not only the lignin content but also its distribution play a role in acting as a physical barrier, affecting polysaccharide accessibility [86]. For instance, when lignin covers the surface of cellulose fibers, accessibility to hydrolytic enzymes is inhibited, therefore reducing the cellulose degradation rate. While retrograded starch/lignin films do not exhibit fibrous morphologies, it is likely that lignin can also cover the surface of crystalline domains of amylose, again acting as a physical barrier, limiting amylolytic enzyme accessibility. Such a mechanism was proposed by Majeed et al. [70], in which 5–20% of kraft lignin (MW = 10 kDa) was incorporated in urea cross-linked starch films. The final application of the films was a slow-release fertilizer. 

**Figure 7:**
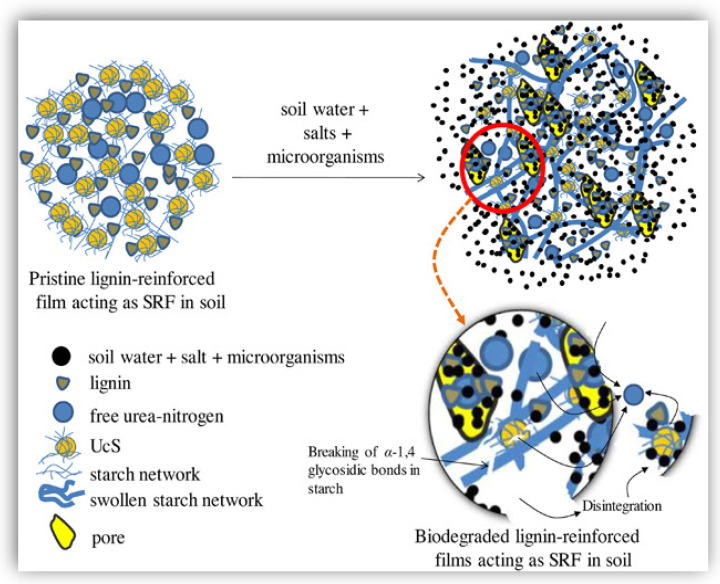
The slow-release fertilizer’s biodegradability due to urea-crosslinked starch films is influenced by the lignin macromolecule. Lignin macromolecule’s implication in slowing the biodegradability of urea-crosslinked starch films applied as slow-release fertilizer. Reprinted with permission from Majeed et al., 2017 [70]. Copyright, 24 July 2024 John Wiley and Sons, License No. 5835691379483.

After the production of lignin-reinforced, cross-linked starch films, they were subjected to biodegradability studies via aerobic soil burial tests for up to 60 days, similar to aerobic composting. Based on the results of FTIR spectroscopy, TGA, and morphological analyses of biodegraded films, the authors proposed a mechanism of lignin protection of starch in lowering the degradation rate (shown in Figure 7). 

First, it is known that functional groups of lignin, such as phenolic -OH groups, form hydrogen bonds with starch hydroxyl -OH groups. Second, and as discussed above, lignin acts as a non-productive binding site for hydrolytic enzymes, mediated by hydrophobic, electrostatic, and hydrogen-bonding interactions. Therefore, the authors proposed that lignin, in addition to acting as a physical barrier limiting accessibility of starch to amylases, causes steric hindrance to enzymes through non-productive binding. The final result is limited diffusion of amylases within the film lignin/starch structure and a lower rate of degradation. Overall, lignin offers multiple characteristics that improve the degradation properties of starch films, including hydrophobic (aromatic) groups and hydrophilic (charged) groups, which provide both a physical barrier and sites for non-productive binding of amylolytic enzymes via a unique combination of hydrogen-bonding, hydrophobic (van der Waals), and electrostatic interactions. 

## 6. Materials and Methods of Characterization of Polysaccharide Films with Lignins

In the majority of cases, the fabrication of the starch films with lignin follows a conventional methodology that involves the preparation of lignin (purification), its dilution in the solvent, and its incorporation into the starch solution. The most frequently utilized solvents are distilled water, dimethyl sulfoxide (DMSO), dialysis tube, plasticizer (glycerol), and Teflon film (or Teflon film-coated glass plate). According to Tian et al. [62], to purify the lignin, it should be dissolved in DMSO to form a homogeneous solution (e.g., 400 mg of lignin in 200 mL of the solvent). One should put the resultant lignin solution into a dialysis tube. Up until there is no longer any detectable DMSO in the wastewater, dialysis is carried out using more water than the trap and is repeated on a regular basis. Once the concentration of the obtained lignin particle dispersion has been adjusted to 4 mg mL^−1^ by evaporating excess water, it is finally stored in a refrigerator (4 °C) for additional characterization.

In Shankar et al. [66], prior to fabricating the films, the lignin was dried at 80 °C for 6 h. Using a magnetic stirrer, the necessary quantity of lignin was mixed with 150 mL of distilled water and stirred for two hours. Next, using a hot plate, the suspension was vigorously stirred while the amount of polysaccharide and glycerol was added. This process was done for 30 min at 95 °C. A leveled, 24 cm by 30 cm glass plate coated with Teflon film was filled evenly with the fully soluble film-forming solution, and it was left to dry for approximately 48 h at room temperature. Before conducting an experimental analysis, the dried films were removed from the plate and allowed to condition for 48 h at a temperature of 25 °C and a relative humidity of 50%.

Hereafter, a description of the necessary tests to assess the effect of lignin in the composite films is given.

### 6.1. Barrier Properties

The water hold capacity (WHC) test measures the quantity of water that a sample of the film can retain during a specific period of time. The assessment can be conducted by the sieve shake method, where an amount of the film (around 20 mg) is immersed in distillated water for 24 h to ensure swell-up. Then, with the help of tweezers, the film is taken out and put on a 40-mesh sieve. The water on the surface is removed by shaking the sieve twice, and the weight of the wet film is registered. The information obtained is reported in measurements of gram of retained water/gram of dry film [62]. Ul-Islam, Khan, and Park [87] found a direct relation between the WHC, the porosity, and the surface area of films of bacterial cellulose (BC) modified by the addition of chitosan and montmorillonite. As a result, the WHC rose as the films’ pore volume and pore size increased, depending on the kind and arrangement of the composite materials on the BC sheets’ surface and in their matrix. Meanwhile, Tian et al. [62], in their work about composite films of bacterial cellulose reinforced with lignin nanoparticles, demonstrated that WHC also depends on the chemical compositions and structures of the lignin precursor. 

**Figure 8:**
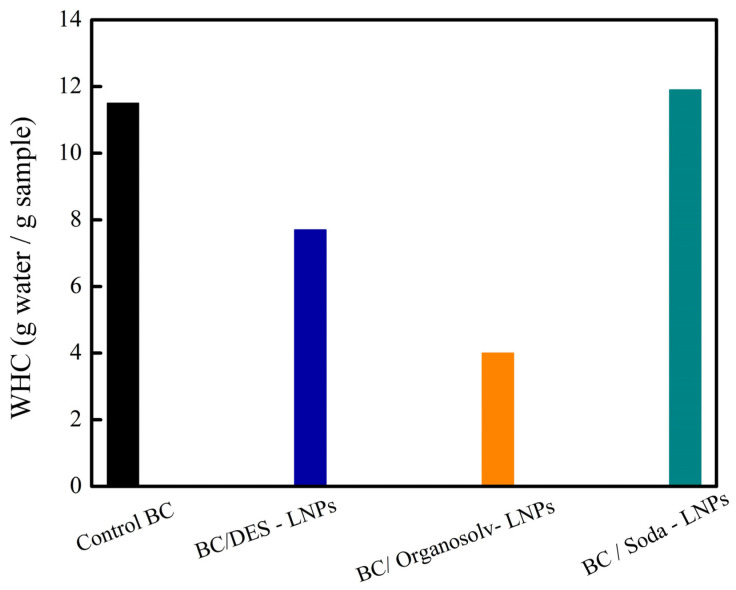
Effect of the LNPs from DES, organosolv, and soda lignins on the WHC of bacterial cellulose composite films. The figure was built with experimental data by Tian et al., 2021 [62]. Copyright, 24 July 2024 Elsevier license No 5835701089403.

Figure 8 compares the WHC of 11.5 of the control film (BC control, without lignin) with the composite films BC/DES LNPs, BC/organosolv LNPs, and BC/soda LNPs, of which the LNPs from DES, organosolv, and soda, respectively, were in situ introduced into BC. The authors explained that the small pores of BC/soda LNPs and the fact that soda lignin provided more carboxyl groups to the BC film were the reasons for the highest WHC value of 11.9. Additionally, soda lignin had increased hydrophilicity, which affected the BC/soda LNPs’ composite films’ WHC. Conversely, the larger particle sizes of the LNPs, which occupied part of the pores in the BC network, were the cause of the lower WHT values of the BC/DES LNPs’ (7.7) and BC/organosolv LNPs’ (4.0) composite films.

#### 6.1.1. Water Vapor Permeability (WVP)

In starch-based films, water vapor permeability is a crucial parameter that indicates how well the film can control the movement of water vapor between food and its surroundings. Starch-based films typically have higher WVP and are not very good at preventing water vapor [67]. Particularly in a moist environment, the abundant intrinsic hydroxyl groups have the ability to adsorb large amounts of water, weakening the corresponding strength and breaking the starch film [37]. This constitutes one of the main disadvantages for its commercial applications as packaging material. The protocol of the test is based on the standards ASTM E96-95, but some modifications have been introduced according to the needs [67,88,89].

As an example, the WVP data of sago starch/alkali lignin films were determined according to the procedure as follows. Firstly, five distinct locations on the films were used to measure their average thicknesses. Next, 15 mL of distilled water (100% RH) was placed in glass permeation cups (4.6 × 2.9 cm) with an exposed film area of 1.66 × 10^−3^ m^2^. Using parafilm, the samples were sealed onto the permeation cup. The sealed cups were sequentially put in desiccators with saturated sodium bromide solution (58% RH) at 30 °C. The weight of the permeation cups was recorded every hour for eight hours after reaching a steady state (<two hours). Ultimately, the weight loss noted in each test cup was used to compute the film’s WVP [67]. The test must be conducted in triplicate samples, and the relation of Equation (1) was used for the calculation, where WVTR (g/h m^2^) is the water vapor transmission rate, which is calculated from the slope of the straight line (g/h) divided by the test area (m^2^); S is the saturation vapor pressure of water at the test temperature (in kPa); R_1_ is the RH in the permeation cup; R_2_ is the RH in the desiccator; and d is the film thickness (m).

(1) $$\mathrm{WVP}\;\left(g\frac{\mathrm{mm}}{m^{2}}h\;\mathrm{kPa}\right)=\left[\frac{\mathrm{WVTR}}{S\left(R_{1}-R_{2}\right)}\right]\times d$$

**Figure 9:**
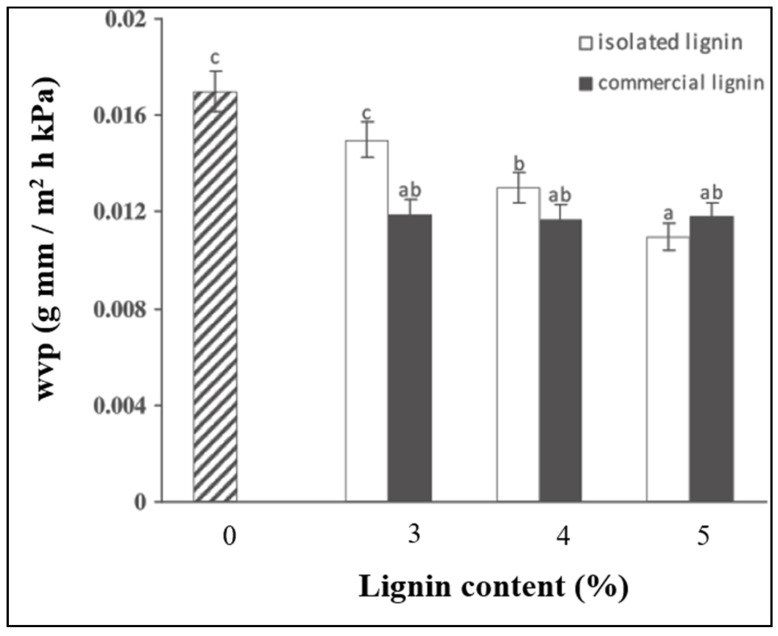
Effect of alkali lignin on the WVP permeability of sago-starch/alkali lignin films. Reprinted with permission from Bhat et al., 2013 [67]. Copyright, 30 July 2024 Elsevier No. 5838970804070.

Figure 9 shows an example of the improvement in the WVP of the film (reduction of the WVP values), which was attributed to strong intermolecular interactions occurring between starch and lignin molecules. 

#### 6.1.2. Water Solubility

In starch-based films, water solubility is a crucial factor; films with high solubility are indicative of low water resistance. The solubility of biopolymer-based films is an essential indicator in determining their potential applications where it is necessary to improve the products’ overall shelf-life, moisture barrier qualities, and integrity. The solubility in water is determined through simple procedural stages, in samples of the film that are cut into small pieces of regular geometry (circular or rectangular) and hydrated and shaken during a specific period of time. Then, the solubility is calculated gravimetrically, considering the oven-dried masses of the sample before and after the hydration [18,90]. In the work of Ramos da Silva, Velasco, and Fakhouri [18], the water solubility of the rice-based starch films was determined through tests in triplicate using disks of 2 cm in diameter, which were previously dried at 105° C for 24 h and weighed. The dehydrated samples were then kept under mechanical agitation (75 rpm) for 24 h at 25 °C after being individually submerged in 50 mL of distillated water. Following this time, the samples that had not yet solubilized were taken out and dried at 105 °C for 24 h. The percentage of solubility was then computed using Equation (2).

(2) $$\mathrm{Solubility}\;\left(\%\right)=\frac{\left(\mathrm{Initial}\;\mathrm{dried}\;\mathrm{mass}\;\mathrm{of}\;\mathrm{the}\;\mathrm{sample}-\mathrm{Final}\;\mathrm{dried}\;\mathrm{mass}\;\mathrm{of}\;\mathrm{nonsolulized}\;\mathrm{sample}\right)}{\mathrm{Initial}\;\mathrm{dried}\;\mathrm{mass}\;\mathrm{of}\;\mathrm{the}\;\mathrm{sample}}\times 100$$

#### 6.1.3. Contact Angle and Surface Energy Test

An easily readable indicator of the surface adhesion between the solid and the liquid drop is the contact angle of a liquid dosed on a solid surface. The ASTM D 5964 standard addresses the procedure to determine contact angle values. Usually, five droplets of the liquid (water in general) at room temperature are gently placed on the film surface, and then the images are captured and recorded. The assessment implies the analysis of an image of the liquid drop on the surface by using various projection or reflective devices and measuring the height and width on the substrate surface. The contact angle is calculated according to Equation (3), and the surface energy can be calculated by the Owens-Wendt-Rable-Kaelble (OWRK) method, using Equations (4) and (5) [91].

(3) $$\theta \;\left(contact\;angle\right)=2\cdot arc\tan\frac{height\;of\;a\;droplet^{\prime }s\;image}{half\;its\;width}$$

(4) $$\sigma_{l}\left(1+cos\theta \right)=2\left(\sqrt{\sigma_{s}^{P}\sigma_{l}^{P}}\times \sqrt{\sigma_{s}^{D}\sigma_{l}^{D}}\right)$$

(5) $$\sigma =\sigma^{P}+\sigma^{D}$$

where the parameters $\sigma^{P}$ and $\sigma^{D}$ represent the polar and dispersive parts, respectively, of the film’s surface energy.

**Figure 10:**
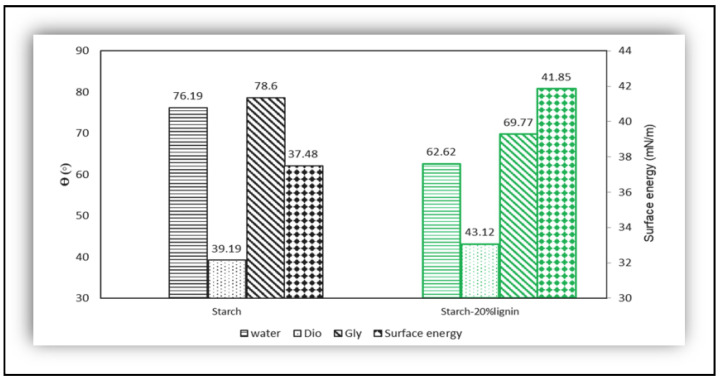
Effect of 20% LNPs in the CA of water, diiodomethane (Dio), and glycerol (Gly) on starch film. Reprinted with permission from Roostazadesh et al., 2022 [92]. Copyright, 30 July 2024 Elsevier No. 5838601217527.

Regarding biopolymer films, the contact angle test can be used for calculating the surface energy and predicting the wettability of films [37,92]. An interesting and non-conventional result was registered by Roostazadesh et al. [92]. As the lignin plays a role in plant cell walls in creating a hydrophobic structure, it should be expected that the addition of nanoparticles of soda lignin in the starch-20% lignin film would increase the contact angle, which did not occur. In Figure 10, the contact angle of pure starch (using water and glycerol as a solvent) decreased from 76.2° and 78.6° to 62.62° and 69.77, respectively. The high concentration of charged phenolic and carboxyl groups during the extraction process is responsible for the chemical changes that the authors attribute to the hydrophilic surface of soda lignin. Another reason was the size of the lignin particles, with more surface area, which could enhance the hydrophilicity of starch film.

**Figure 11:**
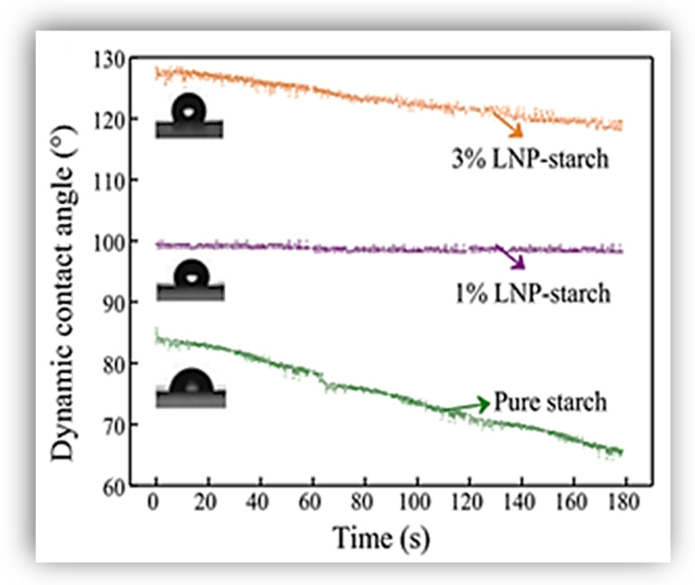
Effect of LNPs in the DCA of LNPs/starch films intended to be used as packaging materials. Adapted with permission from Ni et al., 2022 [37]. Copyright, 30 July 2024 American Chemical Society.

On the other hand, the work of Ni et al. [37], which compared the dynamic contact angle (DCA) (at measurement time of 180 s) of pure starch and its films with 1% and 3% LNPs (from organosolv lignin) demonstrated that lignin significantly improved the DCA of the starch. Figure 11 shows that DCA values of 3% LNP-starch and 1% LNP-starch are maintained at 118° and 98°, respectively, until the end of the test, while the initial 85° of pure starch film sharply decreased until 65° at 180 s. As mentioned in Section 4, the authors ascribed this behavior to the formation of a tight hydrophobic layer due to the cluster aggregation of LNPs. Thus, the LNPs’ cluster aggregations enhanced the surface coarseness of the films from 2.28 nm to 4.84 nm (of 1% LNP-starch film) and 9.97 nm (of 3% LNP-starch film), which increased the hydrophobicity of the films.

#### 6.1.4. Color and Opacity 

In packaging films, in particular, opacity plays a crucial role in overall appearance and consumer acceptance. A calorimeter calibrated to standard black and white tiles is used in the analysis protocol. The mean and standard deviation of the film are then recorded for up to five distinct segments. Several parameters of the color scale should be measured to determine the chromaticity of the film, such as luminosity, range of black to white, and range of blue to yellow. On the other hand, to determine the opacity, a spectrophotometer can also be used, where the samples, previously cut into rectangles, are directly placed into the cuvette, and its absorbance is compared with the reference (an empty cuvette) [18,40].

**Figure 12:**
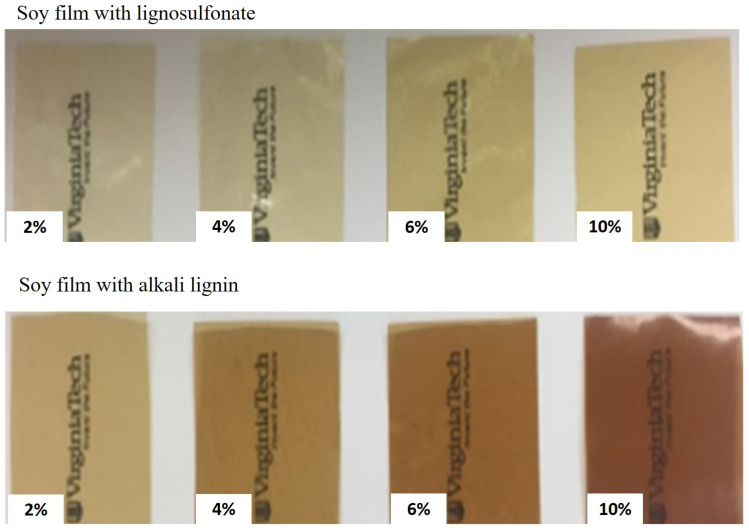
Soy protein isolate films with several concentrations of lignosulfonate and alkali lignin (2, 4, 6, and 10 g of lignin/100 g of soy protein). Adapted with permission from Zadeh, O’Keefe, and Kim, 2018 [40]. Copyright, CC-BY license, 30 July 2024 ACS Publications.

Figure 12 shows the appearance of the enzymatically modified soy protein-based films with different concentrations of lignosulfonate and alkali lignin by Zadeh, O’Keefe, and Kim [40]. The greenness and yellowness increased in the films as a function of lignin concentration. The opacity followed the same trend. In addition, the films with alkali lignin were darker (reddish) than those with lignosulfonate, which is due to the chromophoric nature of lignin that is associated with UV radiation blocking.

### 6.2. Mechanical Properties

Tensile strength (TS), elongation at break (EB), and elastic modulus (EM) are fundamental tests that must be performed in films to define their integrity to stress conditions. As was noticed in most of the consulted works, in starch-lignin films, the responses of these three assays are highly sensitive to any improvement that lignin would add. Regularly, the properties are determined according to the standard ASTM D882, using the universal testing machine with a 100 N load cell and extension speed of 10 mm/min [92].

**Figure 13:**
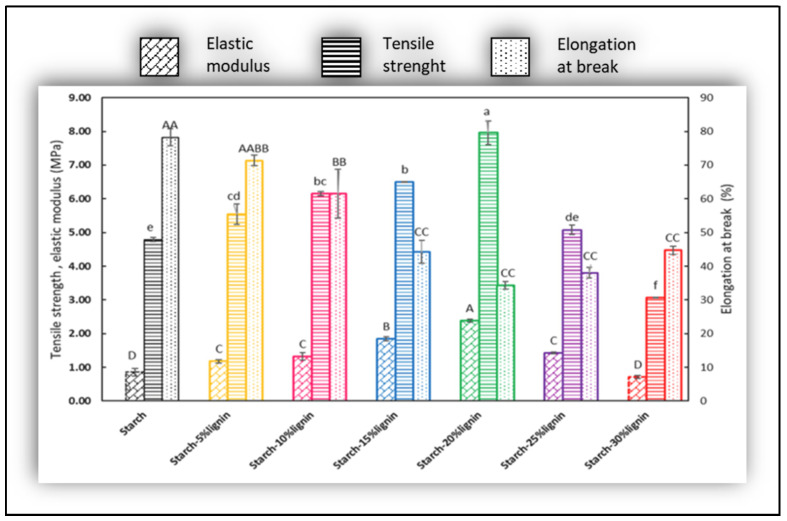
Impact of lignin content and presence on the starch/lignin films’ mechanical performance. Reprinted with permission from Roostazadesh et al., 2022 [92]. Copyright, 30 July 2024 Elsevier No. 5838601217527.

A representative result of the effect of soda lignin in the mechanical performance of the starch-lignin films is shown in the bar chart of Roostazadeh et al. [92] in Figure 13. It is evident that there are significant increases in TS and EM, while the EB decreases. The justification, which is supported by a number of studies, is that the lignin particles establish strong hydrogen bonding and physical interaction with starch polymer chains. Another argument to be considered is that soda lignin is a sulfur-free lignin and thus does not cause degreasing of the flexural properties of the film like what happens when the kraft lignin is used as a reinforcement of the blend [93]. As is exhibited in Figure 13, the higher the lignin content, the higher the tensile strength, except at lignin contents above 20%. Thus, there is a threshold of lignin addition from which mechanical performance decreases due to the agglomeration of lignin particles in the starch matrix [94].

### 6.3. Thermal Properties

Both differential scanning calorimetry (DSC) and thermogravimetric analysis (TGA) have been used to study thermal behavior, thermal stability (T_onset, T_max, T_final, and Char_600), the glass transition temperature of lignins, and even the presence of impurities therein. In addition, numerous works have used thermal properties to assess the miscibility of lignin and its blends. Thermal properties change slightly depending on the precursor and the extraction method. However, for analyzing each film or blend, the specific test conditions must be defined. It is typical to start with a slow heating and cooling cycle to eliminate the thermal history of the sample. At the third cycle of heating/cooling, the thermal events are recorded, in an inert atmosphere, from (25–200) °C. 

**Figure 14:**
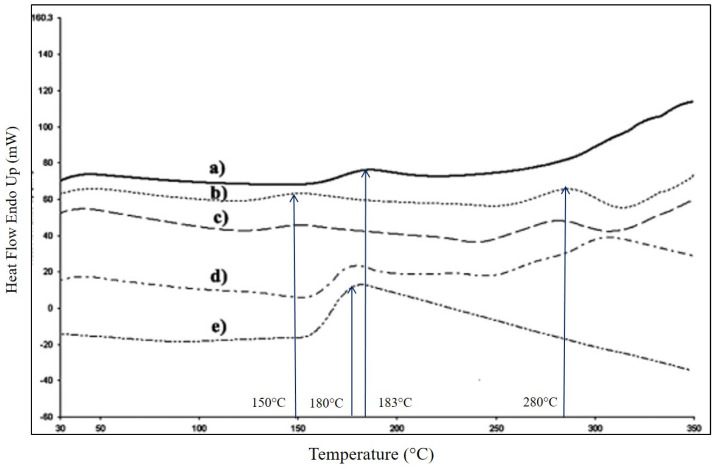
Impact of lignin on the starch/lignin films’ thermal characteristics. (**a**) TPSL0, (**b**) TPSL1.2, (**c**) TPSL1.6, (**d**) TPSL2.0, (**e**) TPSL2.4). (Reprinted with permission from Çalgeris et al., 2012 [94]. Copyright, 30 July 2024 John Wiley and Sons, License No. 583861050189).

Çalgeris et al. [94] studied the effect of the lignin concentration on the crystallinity of starch/alkali lignin films. At low lignin concentration (1.6 to 2.0%), the tensile modulus was high due to the strong interactions between the functional groups of lignin and starch, which generated a strong miscibility of the phases. However, at superior lignin concentrations (as of 2.4%), the modulus decreased sharply because of the phase separation between the starch (hydrophilic) and lignin (hydrophobic) and specifically because of the reduction of the crystalline phase of the films. This behavior could be verified in the heat flow curve of the film without lignin (a) TPSL0 in comparison to those ones where the lignin was added, (b) TPSL1.2, (c) TPSL1.6, (d) TPSL2.0, (e) TPSL2.4, in Figure 14. The curve of the neat starch film showed a melting temperature as 183 °C; however, when the lignin was added, two more endothermic peaks, at around 150 °C and 280 °C, were evident, which demonstrated a strong interaction between the functional groups of starch and lignin. The authors attributed these peaks to the glass transition and melting temperature of lignin, respectively. Conversely, the large endothermic peak in TPSL2.4 at 180 °C was attributed to the melting temperature of the lignin in the film, and the presence of this revealed that lignin suffered deformation of the crystalline phase, i.e., the lignin in the film transformed into an amorphous phase from its semi-crystalline structure. According to these results, it was concluded that there is a threshold for the amount of lignin that should be used in the preparation of starch/lignin film in order to preserve the crystalline phase of lignin and therefore the mechanical properties of the films. 

Anagnou et al. [95] used the DSC technique to characterize the thermal properties of modified softwood kraft lignin with phthalic anhydride (PA SKL) and its blends with poly(ethylene oxide) (PEO) and blends of SKL/PEO. The target of this work was the conversion of lignin to spinnable precursors for producing carbon fibers. Thus, the melting temperature (Tm) and Tg as a function of the ratio lignin/PEO were monitored. The authors related the increase in Tg and reduction of the Tm values as the lignin content increased. This was observed in the flow curves, specifically in PA SKL/PEO blends of 50/50 and 20/80 ratios. These events were related to the modification of the lignin and its influence on the crystallinity percentage and crystalline quality, which enabled the spinning of these blends.

**Figure 15:**
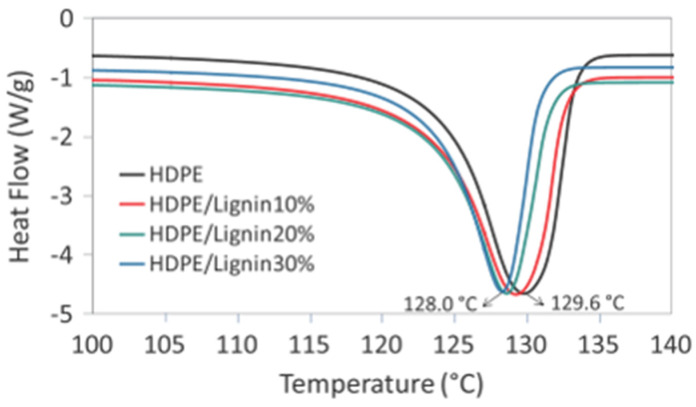
Flow curves of HDPE and HDPE/lignin blends. Reprinted with permission from Sameni, Jaffer, and Sain, 2018 [93]. Copyright, 30 July 2024 Elsevier No. 5838980606723.

Sameni, Jaffer, and Sain [93], in their study, focused on applications in the packaging and automotive industries by monitoring the melting and degradation temperatures of blends of non-wood soda (sulfur-free) lignin with high density polyethylene (HDPE). The flow curves in Figure 15 show a low decline of 1.23% in the melting temperatures of neat HDPE with respect to their blends of HDPE/lignin at 30%. With the addition of lignin, the tensile strength and tensile modulus increased dramatically to 8.6% and 66.0%, respectively. It was determined that these outcomes were caused by soda lignin and HDPE’s improved compatibility, which may be brought about by soda lignin’s low hydroxyl content, low molecular weight, low polar component, and low sulfur. Moreover, the char in the blend was increased by the addition of lignin, which reduced the combustion rate of the polymers.

### 6.4. Biodegradability

The purpose of the biodegradability test is to assess the biodegradability of a given material exposed under humid conditions. Hydrolysis by microorganisms and enzymes is reported to be the most effective method to evaluate this property, especially in composites of bacterial cellulose with lignin [43,62,96]. In enzymatic hydrolysis, the composite with lignin is incubated in a buffer solution at a particular temperature during a period of time of 72 h, as a minimum. Next, a liquid sample of supernatant is collected to measure the amount of glucose produced through HPLC. The calculation of the hydrolyzability of the composites is done by gravimetry, considering the concentration of glucose in the hydrolysate, the volume of the hydrolysate, and the mass of the composite. Tian et al. [62] described how the capability of lignin to limit enzymatic hydrolysis depends on its chemical structure. Thus, lignins with high amounts of phenolic –OH (as DES and organosolv) contributed to the formation of hydrogen bonds with cellulases, which are derived from the inhibition of enzymatic hydrolysis. 

### 6.5. Fourier-Transform Spectroscopy (FTIR)

**Figure 16:**
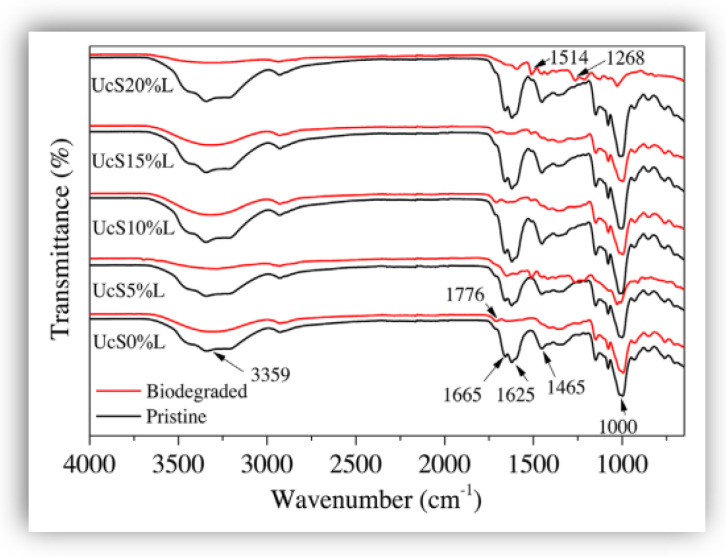
FTIR spectrum of lignin-reinforced films, pristine and biodegraded in aerobic soil. (Reprinted with permission from Majeed et al., 2017 [70]. Copyright, 24 July 2024 John Wiley and Sons, License No. 5835691379483.

The Fourier-transform spectroscopy test allows the determination of changes in functional groups of lignin-reinforced films. Specifically, through this technique, the intensity and the amplitude of the bands in starch-lignin films, which are subject to biodegradation trials, can be monitored. As an example, the change in the intensity of particular peaks in the spectrum of Majeed et al. [70] in Figure 16 allowed the monitoring of biodegradation of urea-crosslinked starch films reinforced with kraft lignin. The black lines are the spectra of starch-urea-lignin films before biodegradation (pristine), and the red lines are the spectra after the trials of biodegradation (soil burial test). 

All the spectra of biodegraded films (red lines) showed an attenuation of the highlighted bands in relation to the pristine films (red lines) due the event of biodegradation. Particular band changes were identified by the authors as the peaks of 1776 cm^−1^ and 1000 cm^−1^, which diminished their intensity as a result of the stretching of the ester carbonyl group in starch after -OH group oxidation by the activity of the natural microorganisms in the soil. Moreover, at 1465 cm^−1^ (in red lines), which corresponds to the asymmetric bending of CH_2_, the reduction in intensity and amplitude was the result of the faster biodegradation in the starch structure than in the lignin. In addition, the intensity of the peaks at 1625 and 1665 cm^−1^ diminished in the biodegraded film without lignin due to fast biodegradability of starch as compared with biodegraded lignin-reinforced films.

### 6.6. Scanning Electron Microscope (SEM)

The scanning electron microscope allows the observation of microstructures and dispersion of lignin into the biopolymer film. Through SEM images, Ni et al. [37] analyzed the roughness of starch/1% LNP films and proposed a mechanism of enhancing hydrophobicity. 

**Figure 17:**
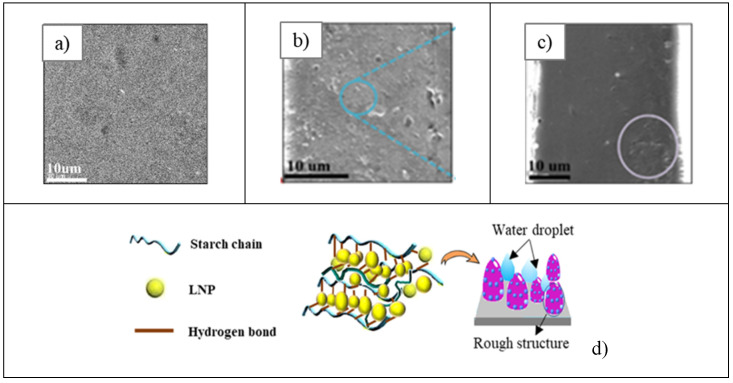
SEM images of surface (**a**,**b**) of the pure starch film and starch-1% LNPs film, respectively; (**c**) cross section of the starch-1% LNPs film with microclusters; (**d**) illustration of the distribution of LPNs in the starch film and the formed possible rough structure. Adapted with permission from Ni et al., 2022 [37]. Copyright, 30 July 2024 American Chemical Society.

Figure 17 shows that the surface of pure starch is smooth (Figure 17a) in comparison with the rough surface of the starch-lignin film (Figure 17b). This indicates that the LNPs aggregated and dispersed in the starch matrix during the solution cast process, as is highlighted with a blue circle in (Figure 17b). However, it is common for some regions of microclusters of LNPs in the starch film to be evident as a result of intermolecular hydrogen bonds, which are formed when the water molecules evaporate during the solution casting process (highlighted in the purple circle in the (Figure 17c) image). In the starch film, the LNPs are organized between the starch chains, which are fixed through hydrogen bonds, building a tridimensional structure as illustrated in the design in (Figure 17d).

### 6.7. X-ray Diffraction (Power XRD or Wide-Angle X-ray Scattering WAXS)

**Figure 18:**
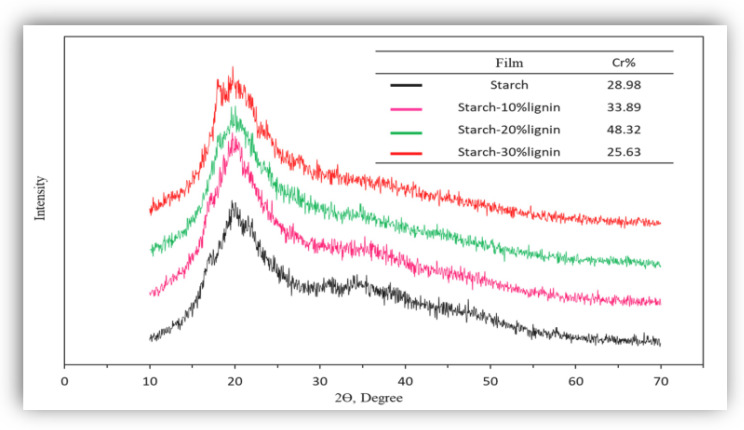
Improvement of the crystallinity of starch-lignin films with the addition of up to 20 wt% of soda lignin. (Reprinted with permission from Roostazadesh et al., 2022 [92]. Copyright, 16 June 2023 Elsevier No. 5570960318321).

Since the X-ray diffraction test is a tool for examining the morphology of thermoplastic starch structures, it is crucial for supplying data regarding the interactions between lignin and polysaccharides. Roostazadeh et al. [92] isolated lignin-rich particles (from wheat straw through alkaline pretreatment) that were used to reinforce starch composite films. It could be observed in the XRD spectra in Figure 18 that after adding 20% lignin particles, the crystallinity of the film enhanced from 29% to 48.3%, and, as a consequence, the tensile strength and modulus increased from 4.8 to 8.0 and from 0.9 to 2.4 MPa, respectively, due to the presence of more nucleating sites. Nevertheless, the addition of more than 20% of lignin goes between starch molecules chains, decreasing its crystallinity. This was attributed to the agglomeration of lignin particles.

Similarly, changes in the crystallinity of polylactic acid (PLA) as lignin was included in the formulation were observed by Tanase-Opedal et al. [97]. PLA exhibited a broad peak at 2θ degrees = 10°–25° associated with its semicrystalline nature. However, with the incorporation of lignin into the matrix, peaks appeared at 2θ = 32° and 34.5°, which indicated greater crystallization of PLA due to the action of lignin as a nucleating agent. This behavior may be due to an increase in interaction due to the increase in bonds between lignin and the matrix and the mechanical interlocking between them [98].

## 7. Conclusions, Challenges, and Perspectives

In terms of valorization of residual agricultural and wood biomass and, in particular, in the improvement of properties of natural polymers, lignin is an attractive solution, as its amphiphilic nature gives rise to desirable properties. 

According to current literature on starch composite films, lignin is an excellent alternative for improving biodegradability and also for introducing new properties. In addition, it has been demonstrated that regardless of the source (wood or herbaceous) or the extraction route (mechanical, conventional pulping chemical, enzymatic, or organosolv), all types of lignins reduce the kinetics of biodegradability of biopolymeric composites. However, the chemical composition and the conformation of the lignin particles play an important role in the action of lignin in the composites.

According to the chemical composition, lignins with lower percentages of aliphatic hydroxyl groups and higher percentages of phenolic hydroxyl groups in the syringyl and guaiacyl units tend to be more repellent to water because they have a greater amount of hydrophobic methoxy groups.

With respect to the conformation of the lignin particles in composites, it has been shown that the introduction of lignin, in the form of nanoparticles in starch films, is the most efficient configuration to improve the biodegradability of these products. This is because each LNP presents a “core-shell” spherical form with the hydrophobic core constituted of methoxy groups and its hydrophilic surface a compound of phenolic and aliphatic hydroxyl groups, which easily unite in hydrogen bonds, forming microclusters in some areas of the composite. These heterogeneously distributed microclusters provide roughness to the film surface, increasing the water contact angle values. 

The fabrication of LNP involves the control of processing parameters of aggregation kinetics and particle size, such as temperature, relative volumes of dispersed and continuous phase, speed of mixing of the phases, and the addition of surfactants and stabilizers, all of which increase production costs. Potential alternatives to overcome these drawbacks include the following:

(a) Using electrostatic stabilizers (as monovalent and divalent chloride salts) to adjust the particulate size of traditional lignins.
(b) Improving the solubility of the native lignin through the manipulation of the pH of the continuous phase.

Thus, it is expected that future research could focus on some of the following issues:

(a) Improvement of lignin extraction techniques that, in addition to being “green”, allow for the efficient removal of lignin with the purity and identity of native lignin and that allow it to be easily solubilized to facilitate applications with polysaccharides such as starch.
(b) Introduction of refining techniques for the extracted lignin to obtain fractions classified by molecular size, composition, and structural conformation. In this manner, the use of each fraction would be targeted to the appropriate application. The low molecular weight fractions could be used for the manufacturing of films with low mechanical resistance and that are highly digestible (encapsulated or edible food packaging). The higher molecular weight fractions could be used in applications with higher mechanical demands and low biodegradability, such as non-edible food packaging, aiming at their industrial production.
(c) Studies could also be focused on the feasibility of using lignin as a direct compatibilizer and mechanical reinforcement in starch films, considering its amphiphilic characteristics and biocompatibility.

## Figures and Tables

**Table 1:** Predominance of structural units and bonds in hardwood, softwood, and herbaceous lignins according to LigninGraphs and comparison with experimental data [52].

Units and Linkages	Softwood (%)		Hardwood (%)		Herbaceous (%)	
	Sim.	Exp.	Sim.	Exp.	Sim.	Exp. *
H	0.0	0.0	0.0	0.0	3.2	4.0
G	100	100	37.7	37	48.0	46
S	0.0	0.0	62.3	63	48.8	50
β-O-4	68.7	66	79.4	78	73.2	68
β-5	19.2	18	4.2	7	14.2	15
β-β	12.1	16	11.7	15	12.6	17
5-5	0.0	0.1	4.9	0.1	0.0	0.0

Sim.: simulated values; Exp: experimental data by [52]; Exp. *: experimental data by [53,54].

**Table 2:** Examples of lignins used to improve properties of biopolymer films.

Type of Lignin	Biopolymer Films	Improvement Property/Application	Reference
Organosolv lignin (Formic acid)	Composite films based on starch	Improvement of hydrophobicity	Ni et al. [37]
DES lignin nanoparticles * Organosolv lignin nanoparticles # Soda lignin nanoparticles +	Composite films made of bacterial cellulose and lignin nanoparticles	Broadening the application, especially in humid conditions	Tian et al. [62]
LignoBoost lignin	Starch/CNF/lignin	Targeting lignin’s application	Zhao et al. [56]
DES, organosolv, soda/AQ, hydrotrope, and kraft lignins	Cellulose/lignin	Antioxidant activity and UV-shielding property/advance packaging	Guo et al. [43]
Alkali and lignosulfonate lignins	Soy protein isolate (SPI)/lignin film	Improvement of radical scavenging activity	Zadeh et al. [40]
Alkali lignin (commercial)	Agar/lignin films	To extend the shelf-life of the packaged food	Shankar et al. [66]
Alkali lignin (commercial, low sulfonate content)	Lignin/sago starch film	Upgrade of water vapor permeability, water resistance, and seal strength/Food packaging industry	Bhat et al. [67]
BLW lignin Δ			
Alkali kraft lignin (Indulin AT)—Sigma Aldrich	Urea-crosslinked starch/lignin films	To enhance the characteristics of slow nitrogen release in naturally aerobic soil	Majeed et al. [69,70]
Acetosolv lignin (wood) LignoBoost lignin	PLA/lignin composites	Improvement of mechanical and water resistance	Spiridon et al. [29]
Kraft lignin	Wheat gluten/lignin bioplastic	Enhancement of mechanical characteristics and decrease in water absorption	Chantapet et al. [71]

* Extracted from poplar wood (hardwood) by steam pretreatment followed by lactic acid-betaine (a deep eutectic solvent—(DES)). # Extracted from poplar wood (hardwood) by steam pretreatment followed by ethanol solvent. + Extracted from poplar wood (hardwood) by steam pretreatment followed by soda/anthraquinone. Δ BLW lignin: precipitated lignin from black liquor wastes (an aqueous solution of lignin, cellulose, hemicellulose, and organic chemicals, byproduct of oil palm process).

## Data Availability

Not applicable.
